# Genetic evidence for an East Asian origin of Chinese Muslim populations Dongxiang and Hui

**DOI:** 10.1038/srep38656

**Published:** 2016-12-07

**Authors:** Hong-Bing Yao, Chuan-Chao Wang, Xiaolan Tao, Lei Shang, Shao-Qing Wen, Bofeng Zhu, Longli Kang, Li Jin, Hui Li

**Affiliations:** 1 Key Laboratory of Evidence Science of Gansu Province, Gansu Institute of Political Science and Law, Lanzhou, 730070, China; 2 State Key Laboratory of Genetic Engineering and Ministry of Education Key Laboratory of Contemporary Anthropology, Collaborative Innovation Center for Genetics and Development, School of Life Sciences, Fudan University, Shanghai, 200433, China; 3 Department of Archaeogenetics and Eurasia3angle research group, Max Planck Institute for the Science of Human History, Kahlaische Straße 10, 07745 Jena, Germany; 4 Key Laboratory of Forensic Genetics, Institute of Forensic Science, Ministry of Public Security, Beijing, 100038, China; 5 School of Medicine, Xi’an Jiaotong University, Xi’an, 710061, China; 6 Key Laboratory of High Altitude Environment and Gene Related to Disease of Tibet, Ministry of Education, Tibet University for Nationalities, Xianyang, Shaanxi, 712082, China; 7 CAS-MPG Partner Institute for Computational Biology, Shanghai Institutes for Biological Sciences, Chinese Academy of Sciences, Shanghai, 200031, China

## Abstract

There is a long-going debate on the genetic origin of Chinese Muslim populations, such as Uygur, Dongxiang, and Hui. However, genetic information for those Muslim populations except Uygur is extremely limited. In this study, we investigated the genetic structure and ancestry of Chinese Muslims by analyzing 15 autosomal short tandem repeats in 652 individuals from Dongxiang, Hui, and Han Chinese populations in Gansu province. Both genetic distance and Bayesian-clustering methods showed significant genetic homogeneity between the two Muslim populations and East Asian populations, suggesting a common genetic ancestry. Our analysis found no evidence of substantial gene flow from Middle East or Europe into Dongxiang and Hui people during their Islamization. The dataset generated in present study are also valuable for forensic identification and paternity tests in China.

Chinese Muslim populations refer to ten officially recognized Muslim ethnic groups, which are Uygur, Dongxiang, Hui, Bo’an, Kazakh, Kirghiz, Salar, Tatar, Tajik, and Uzbek. The origin of those populations via demic diffusion involves mass movement of people or simple cultural diffusion is a long-going debate.

According to historical materials, Islam was first introduced to China some 1,400 years ago in Tang Dynasty (618–907 AD) by a large number of soldiers, merchants and political emissaries from Arabia and Persia (nowadays Middle East)^1^. Chinese Muslim populations are believed to be decedents of those immigrants. Uygur has already been proven to be a typical admixture of East Asian and European by genome-wide scan^2^. However, Dongxinag, Bo’an, and Hui in Gansu and Ningxia have the common physical features of East Asians (Mongoloid type)^3^,^4^,^5^. Xie and Shan^6^ also detected the genetic similarity between Hui and Han Chinese using two autosomal short tandem repeats (STRs) TH01 and D13S317. The paternal Y chromosomal STR clustering has put Hui of Liaoning and Ningxia into the group of Han Chinese and Tibeto-Burman populations^7^. About 24–30% Y chromosomes of Salar, Bo’an, and Dongxiang belong to East Asian specific haplogroup O3-M122. The Central Asian, South Asian, and European prevalent Y chromosomal lineage R-M17 also comprises 17%, 26%, and 28% of Salar, Bo’an, and Dongxiang, respectively^8^.

The origin of Chinese Muslim populations likely involved massive assimilation of indigenous ethnic groups^9^. But previous studies with limited genetic markers and small sample size have not been able to give a clear answer to the genetic ancestry of those Muslim populations. Therefore, we analyzed 15 autosomal STRs in 652 individuals of Dongxiang, Hui, and the co-resident Han Chinese populations in Linxia, Gansu province to explore the genetic diversity of Chinese Muslims and to test population affinities and the level of admixture. Dongxiang and Hui are typical of contemporary Chinese Muslim communities. The comprehensive comparison of those two populations with worldwide Muslims and non-Muslims will shed more light on the origin of Chinese Muslims.

## Methods

We collected blood samples of 163 and 219 unrelated individuals from two Muslim populations Dongxiang and Hui in Linxia, Gansu province. We also collected blood samples of 270 unrelated individuals from Han Chinese in Linxia for comparison purpose. Our study was approved by the Ethical Committee of Gansu Institute of Political Science and Law. The study was conducted in accordance with the human and ethical research principles of Gansu Institute of Political Science and Law. All individuals were adequately informed and signed their informed content before their participation. For each sample, genomic DNA was extracted according to the Chelex-100 method and proteinase K protocol^10^. 15 most widely used forensic loci were amplified simultaneously using AmpFlSTR Sinofiler PCR Amplification Kit (Applied Biosystems, Foster City, CA, USA) at the D8S1179, D21S11, D7S820, CSF1PO, D3S1358, D13S317, D16S539, D2S1338, D19S433, vWA, D18S51, D5S818, FGA, D6S1043 and D12S391 STR loci. The PCR products were analyzed with the 3500XL DNA Genetic Analyzer and Genemapper ID-X software (Applied Biosystems, Foster City, CA, USA).

Allele frequency, heterozygosity, polymorphism information content (PIC), power of discrimination (PD), probability of paternity exclusion (PPE), and other forensic parameters were calculated using PowerStatesV12 (http://www.promega.com/) and Cervus 3.0^11^. Tests for Hardy–Weinberg equilibrium (HWE) were performed in Arlequin v3.5.1.3^12^ using a likelihood ratio test and an exact test to prevent miscalling STR genotypes or biased sampling. Since the statistical analyses in this study were on the basis of Bayesian-clustering algorithm, raw genotypic data of 13 STRs (excluding D6S1043 and D12S391) from 45 populations (13793 individuals) all around the world were extracted to determine population affinity^13^,^14^,^15^,^16^,^17^,^18^,^19^,^20^,^21^,^22^,^23^,^24^,^25^,^26^,^27^,^28^,^29^,^30^,^31^,^32^,^33^,^34^,^35^,^36^,^37^,^38^,^39^. Average number of pairwise differences, pairwise Fst, Slatkins linearized Fst, and coancestry coefficients were all calculated in Arlequin v3.5.1.3 using genotype data^12^. The detailed population genetic structure was performed using model-based clustering method implemented in Structure 2.3.4^40^,^41^ under assumptions of admixture and correlated allele frequencies. Each run used 100,000 estimation iterations for K = 2 to 8 after a 20,000 burn-in length with several replicates. Posterior probabilities for each K were computed for each set of runs. Graphical display for Matrix plot of genetic distance and population structure were carried out in R statistical software v3.0.2^42^ and Distruct v1.1^43^.

## Results

### Forensic parameter analysis

The genotype data for the three populations Dongxiang, Hui, and Han Chinese was given in Supplementary Table 1. The allele frequency distributions and forensic parameters are listed in Table 1 and Supplementary Table 2. The Ho ranged from 0.688 at CSF1PO locus in Hui to 0.914 at D6S1043 locus in Dongxiang while the He ranged from 0.704 at D3S1358 locus in Han to 0.883 at D6S1043 locus in Dongxiang. In the test of HWE, the genotype frequency distributions showed no significant deviations from expectations except p-value of 0.030 at D19S433 locus in Hui. PIC of all selected loci ranged from 0.652 at D3S1358 in Han to 0.868 at D6S1043 in Dongxiang. The values of DP were in the range of 0.861 at D3S1358 in Han to 0.969 in Dongxiang and Hui. The highest PPE was found at D6S1043 locus in Dongxiang (0.824), with the lowest found at CSF1PO locus in Hui (0.410). The most polymorphic loci in all three populations were highly discriminating, which demonstrates that this set of loci will be useful for forensic identification.

### Interpopulation genetic distances

**Figure 1:**
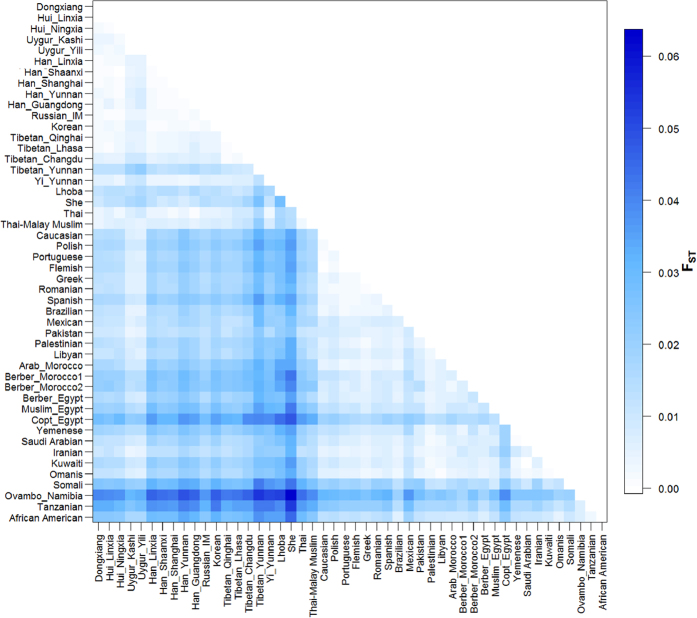
Plots of pairwise Fst of Dongxinag, Hui, and Han Chinese in Linxia and other 45 worldwide populations.

We performed various parameters of genetic distances to infer population structure (Fig. 1 and Supplementary Table 3). Chinese Muslim populations Dongxiang and Hui showed the largest pairwise genetic distances with populations from Africa and Middle East. The smallest genetic distances were noted for Chinese Muslim populations with East Asian populations, especially Han Chinese. Dongxiang showed nonsignificant pairwise Fst difference from Hui in Linxia and Ningxia, Han Chinese in Linxia, Shaanxi, Shanghai, and Guangdong, and Tibetan in Lhasa (p > 0.005). The genetic divergence of Dongxiang and those populations are relatively small (pairwise Fst < 0.002 and Slatkin linearized Fst < 0.003). Hui in Linxia also showed nonsignificant genetic difference with Hui in Ningxia, Uygur in Yili, Han Chinese in Shaanxi and Yunnan, Russian in Inner Mongolia, and Tibetan in Lhasa. Hui in Ningxia also did not differ from all five Han Chinese populations in this study. However, almost all the pairwise Fst differences between Dongxiang and Hui with European, Middle Eastern, and African populations are all above 0.01. The average pairwise differences exhibit the very similar pattern. The two Uygur populations statistically differed from all other populations. The genetic distances of Uygur with East Asian, European, and most Middle Eastern populations are almost the same, indicating Uygur is an admixed population.

### Clustering by structure analysis

**Figure 2:**
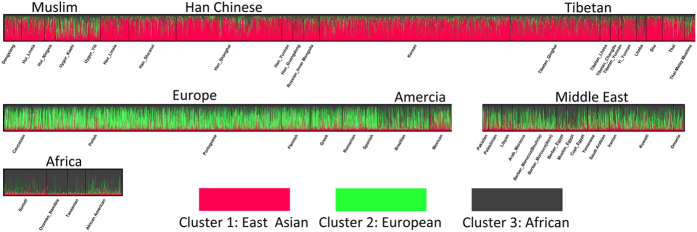
Estimated population genetic structure of Dongxinag, Hui, and Han Chinese in Linxia and other 45 worldwide populations.

Analysis of genetic distance failed to support the genetic affinity between Chinese Muslim populations Dongxiang and Hui with Middle Eastern or European populations. We then employed a cluster based algorithm to further clarify population genetic structure at individual level. According to the highest posterior probabilities, the most suitable K was observed at K = 3 (Supplementary Table 4). The clustering showed a very clear geographic pattern (Fig. 2). East Asian, European, and African populations belong to cluster 1, 2, and 3, respectively. Middle Eastern populations seem like to be admixture of African and European populations. Uygur populations shared a similar degree of membership with East Asian and European (35% to 40%), which is consistent with genetic distance analysis and previous reports^2^. The case of Uygur shows clearly that the 15 STRs and Structure analysis have enough power to delineate the ancestry of populations. The proportion of membership of Dongxiang and Hui in cluster 1 reaches 58.3% to 63.9%. Although this proportion is about 10% lower than Han Chinese (66.8% to 74.4%), it still fall into the general pattern of East Asian populations ranging from 57.8% (Lhoba) to 80.3% (She) (Supplementary Table 4). It’s possible that some individuals of Dongxiang and Hui might have excess affinity with West Eurasians as they are living closely together with Uygur and Central Asians (Supplementary 6). However, the majority of Dongxiang and Hui samples share very similar membership with other East Asian populations, revealing a common genetic makeup.

## Discussion

The origin of Chinese Muslim populations via demic diffusion or simple cultural diffusion has long been a hot debate. Previous genetic studies with limited markers and small sample size often came to contradictory conclusions. In this study, we focused on the genetic makeup and ancestry clustering of Dongxiang and Hui using 15 autosomal STRs genotyped from more than 600 individuals. The two Chinese Muslim populations Dongxiang and Hui showed significant genetic homogeneity with co-resident Han Chinese in Linxia and other East Asian populations rather than with European or Middle Eastern populations, which support a simple cultural diffusion for the origin of Dongxiang and Hui in China. This cultural transformation phenomenon has also been observed in other Muslim populations. Although the Utsat people in Hainan Island are thought to be descendants of the Champa Kingdom and have been officially recognized as Hui nationality, they are genetically much closer to the Hainan indigenous ethnic groups than to the Cham and other mainland Southeast Asian populations^9^. The spread of Islam in the Indian subcontinent was also proven to be predominantly cultural diffusion associated with minor gene flow from West Asia and Arabia by analyzing autosomal STRs^44^,^45^, mitochondrial DNA^46^,^47^, and Y chromosome^47^. Autosomal STRs also reveal common genetic ancestry of the Thai-Malay Muslims and Thai Buddhists^36^. In this context, cultural transformation has shaped worldwide Muslim populations.

## Additional Information

**How to cite this article**: Yao, H.-B. *et al*. Genetic evidence for an East Asian origin of Chinese Muslim populations Dongxiang and Hui. *Sci. Rep.* **6**, 38656; doi: 10.1038/srep38656 (2016).

**Publisher's note:** Springer Nature remains neutral with regard to jurisdictional claims in published maps and institutional affiliations.

## Supplementary Material

Supplementary Information

Supplementary Table 1

Supplementary Table 2

Supplementary Table 3

Supplementary Table 4

Supplementary Table 5

## Figures and Tables

**Table 1:** Forensic statistical parameters of the 15 autosomal short tandem repeats from Dongxiang, Hui, and Han Chinese populations in Linxia.

		CSF1PO	D12S391	D13S317	D16S539	D18S51	D19S433	D21S11	D2S1338	D3S1358	D5S818	D6S1043	D7S820	D8S1179	FGA	vWA
Dongxiang	Ho	0.693	0.853	0.755	0.785	0.810	0.834	0.871	0.822	0.810	0.767	0.914	0.791	0.847	0.847	0.847
	He	0.733	0.844	0.800	0.787	0.868	0.817	0.839	0.868	0.729	0.778	0.883	0.793	0.836	0.860	0.802
	PIC	0.688	0.824	0.767	0.751	0.852	0.791	0.817	0.851	0.680	0.742	0.868	0.758	0.812	0.843	0.769
	DP	0.878	0.952	0.930	0.920	0.965	0.940	0.950	0.963	0.862	0.908	0.969	0.920	0.948	0.962	0.920
	PPE	0.418	0.700	0.518	0.572	0.617	0.664	0.737	0.641	0.617	0.539	0.824	0.583	0.688	0.688	0.688
	NE-1P	0.679	0.478	0.578	0.601	0.424	0.534	0.489	0.428	0.688	0.611	0.392	0.592	0.505	0.441	0.576
	NE-2P	0.502	0.311	0.400	0.423	0.268	0.360	0.321	0.270	0.514	0.432	0.243	0.414	0.333	0.281	0.398
	NE-PP	0.318	0.139	0.219	0.241	0.107	0.178	0.146	0.110	0.334	0.247	0.091	0.233	0.160	0.115	0.219
	NE-I	0.115	0.043	0.071	0.080	0.032	0.058	0.046	0.033	0.121	0.084	0.026	0.076	0.049	0.035	0.071
	NE-SI	0.414	0.340	0.369	0.378	0.325	0.357	0.343	0.325	0.417	0.383	0.316	0.374	0.346	0.330	0.368
	P-Value	0.094	0.738	0.595	0.970	0.313	0.384	0.894	0.172	0.375	0.198	0.755	0.504	0.896	0.623	0.178
Hui_Linxia	Ho	0.688	0.839	0.808	0.776	0.858	0.849	0.831	0.826	0.708	0.749	0.831	0.807	0.817	0.895	0.775
	He	0.728	0.853	0.813	0.785	0.860	0.819	0.835	0.853	0.720	0.777	0.877	0.791	0.817	0.871	0.803
	PIC	0.678	0.835	0.784	0.750	0.843	0.794	0.813	0.833	0.668	0.743	0.862	0.757	0.791	0.855	0.771
	DP	0.877	0.961	0.937	0.914	0.960	0.937	0.951	0.959	0.873	0.916	0.969	0.918	0.938	0.965	0.931
	PPE	0.410	0.674	0.614	0.556	0.710	0.694	0.658	0.649	0.440	0.508	0.655	0.613	0.632	0.785	0.554
	NE-1P	0.690	0.457	0.552	0.601	0.440	0.528	0.496	0.462	0.701	0.608	0.406	0.593	0.538	0.417	0.573
	NE-2P	0.518	0.294	0.375	0.423	0.280	0.355	0.326	0.298	0.530	0.428	0.253	0.415	0.363	0.262	0.395
	NE-PP	0.339	0.126	0.197	0.240	0.115	0.174	0.150	0.130	0.352	0.241	0.098	0.233	0.184	0.103	0.215
	NE-I	0.124	0.039	0.063	0.080	0.035	0.056	0.048	0.040	0.130	0.083	0.029	0.077	0.059	0.031	0.069
	NE-SI	0.418	0.334	0.360	0.378	0.330	0.355	0.345	0.335	0.423	0.383	0.320	0.375	0.357	0.323	0.367
	P-Value	0.344	0.968	0.394	0.100	0.492	0.030	0.287	0.225	0.714	0.564	0.268	0.255	0.969	0.639	0.258
Han_Linxia	Ho	0.741	0.847	0.800	0.748	0.844	0.870	0.802	0.844	0.703	0.785	0.900	0.789	0.830	0.856	0.814
	He	0.735	0.849	0.807	0.775	0.857	0.830	0.824	0.860	0.704	0.766	0.874	0.769	0.832	0.857	0.810
	PIC	0.689	0.830	0.777	0.738	0.839	0.808	0.799	0.843	0.652	0.728	0.859	0.734	0.809	0.839	0.780
	DP	0.883	0.956	0.932	0.915	0.961	0.942	0.945	0.964	0.861	0.904	0.965	0.910	0.947	0.960	0.934
	PPE	0.494	0.689	0.599	0.507	0.684	0.735	0.606	0.684	0.432	0.572	0.795	0.579	0.655	0.706	0.626
	NE-1P	0.676	0.468	0.563	0.619	0.449	0.506	0.512	0.444	0.714	0.632	0.411	0.622	0.507	0.448	0.558
	NE-2P	0.501	0.303	0.386	0.440	0.288	0.335	0.340	0.283	0.544	0.452	0.257	0.442	0.335	0.287	0.381
	NE-PP	0.318	0.134	0.207	0.258	0.122	0.158	0.159	0.119	0.363	0.268	0.101	0.255	0.160	0.120	0.202
	NE-I	0.115	0.041	0.066	0.087	0.037	0.050	0.052	0.036	0.139	0.092	0.030	0.088	0.050	0.037	0.065
	NE-SI	0.412	0.336	0.364	0.385	0.332	0.348	0.352	0.330	0.433	0.391	0.321	0.388	0.347	0.332	0.362
	P-Value	0.954	0.331	0.265	0.543	0.639	0.560	0.523	0.928	0.122	0.618	0.172	0.396	0.191	0.398	0.828
