# Supplementary material for: Genetic evidence for an East Asian origin of Chinese Muslim populations Dongxiang and Hui
Source: Sci Rep. 2016 Dec 7;6:38656. doi: 10.1038/srep38656 (PMC5141421; doi:10.1038/srep38656)

**Article for Scientific Reports**

**Genetic evidence for an East Asian origin of** **Chinese Muslim populations Dongxiang and Hui**

Hong-Bing Yao^1, ✝^,*, Chuan-Chao Wang^2, 3^, ^✝^,*, Xiaolan Tao^1^, Lei Shang^4^, Shao-Qing Wen^2^, Bofeng Zhu^5^, Longli Kang^6^, Li Jin^2, 7^, Hui Li^2^, *

1. Key Laboratory of Evidence Science of Gansu Province, Gansu Institute of Political Science and Law, Lanzhou, 730070, China.
2. State Key Laboratory of Genetic Engineering and Ministry of Education Key Laboratory of Contemporary Anthropology, Collaborative Innovation Center for Genetics and Development, School of Life Sciences, Fudan University, Shanghai, 200433, China.
3. Max Planck Institute for History and the Sciences, Kahlaische Straße 10, 07745 Jena, Germany.
4. Key Laboratory of Forensic Genetics, Institute of Forensic Science, Ministry of Public Security, Beijing, 100038, China.
5. School of Medicine, Xi’an Jiaotong University, Xi’an, 710061, China.
6. Key Laboratory of High Altitude Environment and Gene Related to Disease of Tibet, Ministry of Education, Tibet University for Nationalities, Xianyang, Shaanxi, 712082, China.
7. CAS-MPG Partner Institute for Computational Biology, Shanghai Institutes for Biological Sciences, Chinese Academy of Sciences, Shanghai, 200031, China.

✝These authors contributed equally to this work.

*Corresponding author: Hui Li, Hong-Bing Yao, and Chuan-Chao Wang. Tel: +86-21-51630427, E-mail addresses: [LHCA@Fudan.edu.cn](mailto:LHCA@Fudan.edu.cn) (Hui Li) Tel: +86-931-7601409, E-mail addresses: [yaohongb@126.com](mailto:yaohongb@126.com) (Hong-Bing Yao) Tel: +49 (0) 3641 686-648, E-mail: [wang@shh.mpg.de](mailto:wang@shh.mpg.de) (Chuan-Chao Wang).

**Table S6. Proportion of individuals in each population with >50% membership of each pre-defined cluster in Structure analysis**

| **Populations** | **1** | **2** | **3** |
| --- | --- | --- | --- |
| Dongxiang | 0.681 | 0.104 | 0.110 |
| Hui_Linxia | 0.726 | 0.105 | 0.046 |
| Hui_Ningxia | 0.620 | 0.120 | 0.050 |
| Uygur_Kashi | 0.373 | 0.286 | 0.127 |
| Uygur_Yili | 0.330 | 0.302 | 0.123 |
| Han_Linxia | 0.811 | 0.041 | 0.052 |
| Han_Shananxi | 0.776 | 0.081 | 0.040 |
| Han_Shanghai | 0.807 | 0.058 | 0.038 |
| Han_Yunnan | 0.838 | 0.019 | 0.048 |
| Han_Guangdong | 0.745 | 0.039 | 0.137 |

As we have documented, Cluster 2 and Cluster 3 may represent the West Eurasian enriched ancestry in Structure analysis. We find the proportions of individuals in Dongxiang and Hui with >50% membership of Cluster 2 and Cluster 3 are slightly higher than those in Han Chinese, which suggests some individuals in Dongxiang and Hui might have excess affinity with West Eurasians compared with Han Chinese.

We then carried out a principal component analysis (PCA) on the results of Structure analysis. We find that some individuals of Dongxiang and Hui are shifted towards European and African directions. But we caution that this is not direct evidence for West admixture, since there are also Han Chinese shifted towards Europeans and Africans, and some European and African individuals are plotted towards East Asians. We caution that the forensic STR loci might not be informative at individual level to reveal detailed ancestry components.


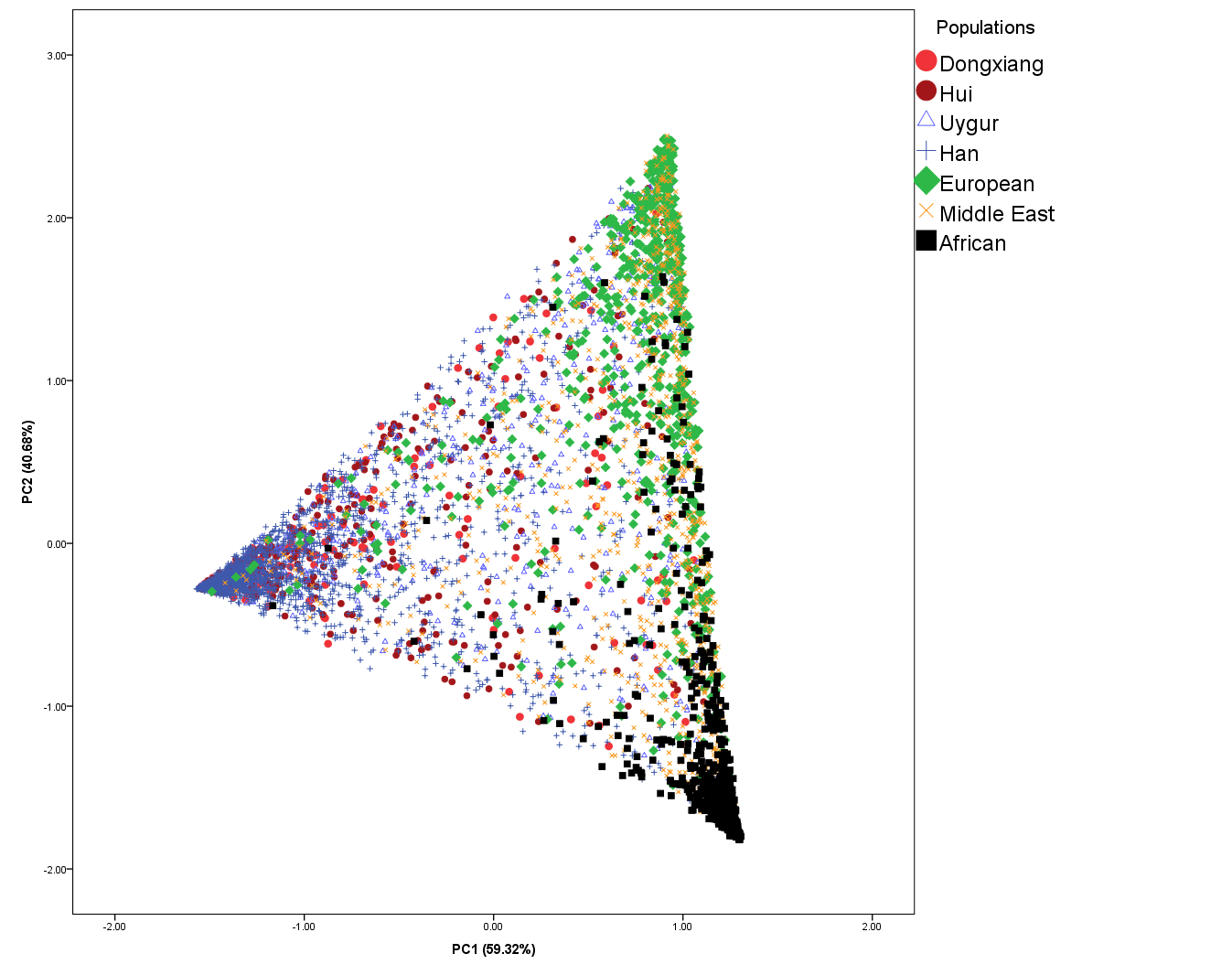

Supplement: Supplementary Information [file srep38656-s1.doc]
